# Persistent cannabis use as an independent risk factor for violent behaviors in patients with schizophrenia

**DOI:** 10.1038/s41537-020-0104-x

**Published:** 2020-05-11

**Authors:** Mélissa Beaudoin, Stéphane Potvin, Charles-Edouard Giguère, Sophie-Lena Discepola, Alexandre Dumais

**Affiliations:** 1 Centre de recherche de l’Institut universitaire en santé mentale de Montréal, Montreal, QC Canada; 2 Faculty of Medicine, Department of Psychiatry and Addictology, Université de Montréal, Montreal, QC Canada; 3 Institut national de psychiatrie légale Philippe-Pinel, Montreal, QC Canada

**Keywords:** Schizophrenia, Human behaviour

## Abstract

Although recent studies have shown a moderately strong association between cannabis use and violence among people with severe mental disorders, the direction of this association has not been investigated prospectively in a population with schizophrenia. Therefore, this study aims to determine, using cross-lag models, whether a temporal relationship between cumulative cannabis use and violence exists in a population with schizophrenia. The authors reported findings covering an 18-month period from a randomized, double-blind clinical trial of antipsychotic medications for schizophrenia treatment. Among the 1460 patients enrolled in the trial, 965 were followed longitudinally. Although persistent cannabis use predicted subsequent violence, violence did not predict cannabis use. The relationship was therefore unidirectional and persisted when controlling for stimulants and alcohol use. Finally, a significant body of evidence suggests a link between persistent cannabis use and violence among people with mental illnesses. Studies to further investigate the mechanisms underlying this association should be conducted.

## Introduction

Cannabis is markedly the most frequently abused illicit drug worldwide. In fact, about 2.5% of the world population consumes cannabis yearly, whereas the annual prevalence of cocaine use, such as that of opiates, is of 0.2%^1^. Many nations around the globe have legalized this substance: in 2017, 20% of the US population resided in a state where adults could freely purchase cannabis^2^. However, the effects of its legalization on public health remain uncertain^3^ and cannabis use involves significant risks on mental and physical health^4^. Thus, heavy and regular cannabis consumption could increase the likelihood of anxiety, depression, suicidal ideation and tendencies, manic episodes, and psychosis^5–7^. Among individuals with mental illnesses, cannabis use is associated with more severe symptomatology^5,8^ and with violent behaviors^9,10^.

Interpersonal violence seriously affects people’s lives by increasing the risk of substance dependence, depression, suicide, unemployment, relationship difficulties, and premature death^11^. Worldwide, every year, more than 1.3 million people die because of violence in all of its forms^11^. A positive relationship between schizophrenia and violent behaviors has consistently been observed^12^. Violence within this population is associated with poor insight^13^, poor impulse control^13,14^, positive symptoms^15,16^, antisocial personality traits (including childhood conduct disorder)^16,17^, depression, anger, anxiety^18^, a history of physical and sexual victimization^15,19^, and previous episodes of violent behaviors^20^. Treatment with antipsychotic medications reduces the risk of violence^21^, whereas treatment nonadherence increases the aforementioned risk^20,22^. Although substance abuse exacerbates violence in schizophrenia^15,23^, very few studies have examined the role of each abused substance separately. Literature on this association with regards to cannabis is especially limited, whereas the effects of alcohol and stimulants on violence are well known^24^.

Results from a recent meta-analysis suggest that cannabis use significantly aggravates violent behaviors among people with severe mental illness, with an odds ratio (OR) of 3.02 (95% confidence interval [CI] = 2.01–4.54, *p* = 0.0001)^10^. This association’s strength increases when the use of cannabis persists through time, as demonstrated in a study previously carried out by our research team, which involved patients with various mental disorders^9^. However, only two studies included in the meta-analysis^10^ focused exclusively on schizophrenia^25,26^: their designs were cross-sectional and their sample sizes were very small (*N* = 70 and 59). Two other studies calculated a cannabis–violence association in early psychosis patients, but neither created longitudinal models to confirm the directionality of this association^27,28^. Therefore, it is also possible that violence precedes cannabis use, as suggested by the results of a few longitudinal studies conducted on general population^29,30^ and on psychiatric populations^31,32^. However, this hypothesis was never tested in a sample constituted exclusively of schizophrenia patients.

Based on findings in populations with severe mental illnesses, we hypothesized that cumulative cannabis use increases the risk of violence across time in a subsample from the Clinical Antipsychotic Trials of Intervention Effectiveness (CATIE) Schizophrenia Trial. Namely, this large randomized high-quality clinical trial has been used to study various issues relating to violence in schizophrenia^17,21,23^. Our study complements previous discoveries, as we used this database to investigate the impact of cannabis on violence over time. Moreover, the unidirectional relationship between persistent cannabis use and violence within a sample of individuals with various severe mental illnesses has been observed^9,10^. We aimed to validate the unidirectional association between cannabis consumption and violence in people suffering specifically from schizophrenia. As such, our study investigate the directionality of this longitudinal relationship in a schizophrenia-specific population.

## Results

### Sample characteristics

In the sample of 965 participants having completed a follow-up visit at 6 and/or 12 months, the average age was of 41.06 years (SD = 11.01; range = 18–67 years old) and the majority were men (72.3%). The mean schooling duration was of 11.59 years (SD = 3.49) and one quarter of participants did not complete high school (25.0%). Only 11.1% were married and 21.1% were veterans. A minority of participants were violent during period 1 (11.5%) and the prevalence of violent acts progressively decreased (6.0% during period 2 and 5.3% during period 3). Complete sample characteristics, including substance use, are included in Supplementary Table 2.

To account for potentially interfering variables, possible violence predictors, measured during the baseline interview, were included in the descriptive analyses. Some variables have been significantly associated with violence as follows: age, educational level, childhood antisocial behaviors, alcohol use, cannabis use, and amphetamine use. The comparisons between the violent and nonviolent groups are presented in Table 1.

**Table 1:** Descriptive analyses.

		Nonviolent at 6 and 12 months^a^ (*N* = 880/965)		Violent at 6 and/or 12 months (*N* = 85/965)		
Continuous variables	Test	Mean	SD	Mean	SD	*p*-Value
Age (*N* = 964)	M–W	41.4	10.9	37.6	11.3	0.005*
Number of years of patient education (*N* = 960)	M–W	11.7	3.47	10.7	3.54	0.001**
Years since first treatment for behavioral or emotional problem (*N* = 937)	M–W	16.7	11.3	15.4	11.2	0.37
Years since first prescribed antipsychotic medication (*N* = 942)	M–W	14.4	11.1	12.8	10.8	0.18
Childhood antisocial behavior severity (*N* = 964)	M–W	1.04	1.37	1.82	1.66	<0.001**
CDS total score (*N* = 963)	M–W	4.42	4.37	5.17	4.31	0.070
ITAQ total score (*N* = 959)	M–W	18.4	4.92	18.3	4.57	0.49
PANSS Positive Subscale Score (*N* = 962)	M–W	18.1	5.51	18.3	5.39	0.46
PANSS Negative Subscale Score (*N* = 962)	M–W	20.4	6.44	19.7	7.08	0.31
PANSS General Subscale Score (*N* = 962)	M–W	36.8	9.29	37.2	9.62	0.74
QLS Total Score (*N* = 957)	M–W	2.76	1.06	2.90	1.20	0.44

Dichotomic variables	Test	No**.**	%	No.	%	*p*-Value
Gender = male (*N* = 698/965)	*χ*^2^	634	72.0	64	75.3	0.52
Being married at the baseline visit (*N* = 107/964)	*χ*^2^	94	10.7	13	15.3	0.20
Currently living with a significant other at baseline visit (*N* = 167/964)	*χ*^2^	146	16.6	21	24.7	0.060
White (*N* = 602/965)	*χ*^2^	555	63.1	46	55.3	0.16
Black (*N* = 326/965)	*χ*^2^	292	33.2	34	40.0	0.20
American Indian or Alaska Native (*N* = 15/965)	*χ*^2^	14	1.6	1	1.2	0.77
Asian (*N* = 27/965)	*χ*^2^	26	3.0	1	1.2	0.34
Hispanic Latino or Spanish Origin (*N* = 111/965)	*χ*^2^	101	11.5	10	11.8	0.94
Hawaiian or Pacific Islanders (*N* = 7/965)	Fisher	5	0.6	2	2.4	0.064
Have been hospitalized or required crisis stabilization in the 3 months preceding the baseline visit (*N* = 236/965)	*χ*^2^	212	24.1	24	28.2	0.40
Obsessive-compulsive disorder (*N* = 43/965)	Fisher	39	4.4	4	4.7	0.79
Other anxiety disorders (*N* = 86/965)	*χ*^2^	78	8.9	8	9.4	0.87
Major depression (*N* = 135/965)	*χ*^2^	118	13.4	17	20.0	0.094
Employed full time at the baseline visit (*N* = 60/956)	*χ*^2^	56	6.4	4	4.7	0.55
Exposition to quetiapine (baseline—12 months) (*N* = 369/965)	*χ*^2^	333	37.8	36	42.4	0.41
Exposition to clozapine (baseline—12 months) (*N* = 81/965)	*χ*^2^	74	8.4	7	8.2	0.96
Alcohol consumption in the past three months (*N* = 334/962)	*χ*^2^	287	32.6	47	55.3	<0.001**
Cannabis consumption in the past three months (*N* = 154/962)	*χ*^2^	121	13.8	33	38.8	<0.001**
Opiates consumption in the past three months (*N* = 9/962)	Fisher	8	0.9	1	1.2	0.56
PCP consumption in the past three months (*N* = 1/962)	Fisher	1	0.1	0	0.0	1.00
Any stimulant (cocaine or amphetamines) consumption in the past three months (*N* = 75/962)	*χ*^2^	66	7.5	9	10.6	0.30
Nonadherence to medication in the past three months following baseline (*N* = 43/389)	Fisher	41	11.2	2	8.7	1.000

*N* = 965.

*CDS* Calgary Depression Scale, *ITAQ* Insight & Treatment Attitudes Questionnaire, *M–W* Mann–Whitney, *PANSS* Positive and Negative Syndrome Scale, *QLS* Quality of Life Scale.

*p ≤ 0.05; **p ≤ 0.001.

^a^Includes those with a missing time-point (at 6 or 12 months) who were nonviolent.

### Cross-lag models

Results of the first cross-lag model are reported in Fig. 3a. Without controlling for possibly confounding factors, it was first observed that violence predicts further violence, and that cannabis use predicts further persistent cannabis use. Most importantly, cannabis use during period 1 predicts violence in period 2 (*p* < 0.001) and cumulative cannabis use during periods 1 and 2 predicts violence in period 3 (*p* = 0.038). In contrast, violence during period 1 and period 2 does not predict further persistent cannabis use, with very small standardized coefficients of 0.031 and −0.020 (*p*-values of 0.07 and 0.27, respectively).

A second cross-lag model was calculated to evaluate the possible interactions with stimulant and alcohol use. Results are reported in Fig. 3b. Alcohol and stimulants consumption during period 1 were associated with cannabis use during period 1 (both *p*-values under 0.001). Alcohol use was also associated with violence during period 1 (*p* < 0.001), but stimulant use was not (*p* = 0.76). In addition, all previously observed associations between cumulative cannabis use and violence remained significant.

Every variable associated with violence at a significant descriptive level (see Table 1) was then included as covariable in a third cross-lag model, presented in Fig. 3c. All previously observed associations between cannabis use and violence remained significant, except for the association between cumulative cannabis use during periods 1 and 2, and violence during period 3 (*p* = 0.08). However, effect sizes were not significantly different from those observed in models 1 and 2.

For cross-lag model 1 to 3, every standardized coefficient and their reporting SEs and *p*-values, including nonsignificant associations, are reported in the Supplementary Table 4.

In addition, identical models evaluating the relationships between alcohol/stimulant consumption and violence were calculated; these are presented in Supplementary Figs 1 and 2. First, alcohol use during period 1 was significantly associated with violence during period 2, even afer adjustment for confounding factors (*p* < 0.05). The reverse relationship (violence predicting persistant alcohol use) was nonsignificant. On the other hand, persistant alcohol use during period 1 and 2 did not predict subsequent violence. Second, stimulant use was not predictive of further violent behaviors and violent behaviors did not predict persistant stimulant use. However, cumulative stimulant use during period 1 and 2 was associated with violence during period 2, but this effect was no more significant after adjustment for alcohol and cannabis consumption at baseline.

## Discussion

This study aimed to confirm the longitudinal association between persistent cannabis use and violence in a sample of patients with schizophrenia. It also aimed to supplement the previously acquired information on violence in the CATIE database by considering cannabis as a separate risk factor from other illicit substances. Although a moderate relationship between cannabis use and violence had previously been found in populations with severe mental illnesses^9,10,28^, no study has yet investigated the directionality of this association in a schizophrenia-specific sample. Using data from the CATIE schizophrenia trial, we were able to build models assessing the relationship between cumulative cannabis use and violent behaviors over time.

A unidirectional relationship between cannabis use and violence was identified in models 1 and 2. Model 1 did not include any covariables, whereas model 2 included alcohol and stimulant use as covariables. Identical models were calculated for alcohol and stimulant consumption; however, only alcohol during period 1 predicted violence during period 2. As models 2 and 3 took into account alcohol use during period 1, the observed effects of cannabis on violence were independent of the consumption of other substances. These results are consistent with previous observations related to cannabis consumption and violence in people with severe mental disorders, as reported in the literature. Yet, our results are specific to a population with schizophrenia, thereby confirming our hypothesis. Indeed, the relationship between substance abuse and violence in individuals with severe mental illnesses^22,33^, and more specifically, with schizophrenia^13,23^, is well known. However, although the roles of alcohol and stimulant consumption on violence have been the subject of various studies, there has been little interest in the effects of cannabis on violence. Still, a recent re-analysis of the MacArthur Violence Risk Assessment Study found that persistency of cannabis use predicts violence (OR = 2.44; CI = 1.06–5.63; *p* = 0.036). This effect on violence was higher than that of cocaine use (OR = 0.59; CI = 0.21–1.63; *p* = 0.304) and similar to that of alcohol use (OR = 2.32; CI = 1.25–4.28; *p* = 0.007)^9^. An association between cannabis use and violent behaviors, with various definitions, was also observed in a few longitudinal and cross-sectional studies^10^, although none investigated the directionality of this longitudinal relationship in a schizophrenia-specific population. A recent meta-analysis found a moderate relationship between cannabis use and violence in people with severe mental illnesses (OR = 3.02; CI = 2.01–4.54; *p* = 0.0001): the effect was significantly higher when comparing cannabis misuse (OR = 5.8; CI = 3.27–10.28; *p* = 0.0001) to simple cannabis use (OR = 2.04; CI = 1.36–3.05; *p* = 0.001)^10^. The same trend between cannabis use and violence was found in model 3, with no significant difference in the standardized estimates across models. This trend was observed despite the marginal statistical significance of the association between cumulative cannabis use (period 2) and further violence (period 3) due to lack of power.

Some suggested hypotheses could explain the relationship between cumulative cannabis use and violence in individuals with schizophrenia. First, it is possible that people with a higher cumulative use of cannabis are at higher risk of cannabis misuse. Such a consequence could be a direct result of acute or chronic cannabis intake, or of cannabis withdrawal syndrome, all of which have been associated with increased hostility, irritability, and anger among the general population^34–37^. Indeed, the literature shows that these three factors have repeatedly been associated with violence in people with severe mental illnesses^38–40^. Second, cannabis intake could also have greater effects on a specific subgroup. For example, cannabis users suffering from severe mental illnesses with higher psychopathy and anger scores, as well as a lower verbal IQ, has been identified to be at higher risk of violence over time^41^. In people with schizophrenia, cannabis use could result in changes in symptomatology^42^, which, in turn, could increase the risk of violence^23^. However, the study’s design rendered it impossible to evaluate the expression of schizophrenia symptoms during cannabis use and withdrawal.

People with schizophrenia are at higher risk of substance abuse and of violence than the general population, with a lifetime rate around 27%^43^. Thus, these results indicate that the use of cannabis should be considered when assessing an individual’s violence risk, in clinical and in legal settings alike. Moreover, these findings should be taken into account when formulating clinical recommendations for patients at high risk of violence. Finally, these observations should be transmitted to the public to raise awareness relating to the potential harmful effects of cannabis.

The CATIE database, resulting from a major study conducted in the United States, provided a very wide range of data from a very large sample, thereby allowing us to effectively investigate the relationship between cannabis use and violence, and to confirm our hypothesis. Nonetheless, there are a few limitations that must be mentioned. First, the dichotomic assessment of substance use did not allow to distinguish between different patterns of use. Therefore, further studies should investigate the link between consumption profiles and violence in individuals with mental disorders. Second, as information from collaterals was only available for less than half of the sample, violence measurements were mostly self-reported. An underestimation of the rates of violence was therefore possible; yet, inconsistencies in which solely the collateral reported acts of violence were observed in a small minority of patients (<3%). The self-report has thus been found to be a reliable assessment of violence in a population with mental illnesses^44^. Third, the occurrence of violent acts was much lower at follow-ups than at baseline. One explanation could be that violent participants were at higher risk of leaving the study (unadjusted OR = 1.53; 95% CI = 1.11–2.10). This could also be a result of treatment efficacy. Due to the low prevalence of violence, violence severity could not be considered in predictive models. Also, the longitudinal design of the CATIE study only allowed the measure of psychotic symptoms every 3 months. It was therefore not possible to assess changes in symptomatology immediately after cannabis consumption or withdrawal. Finally, participants from the CATIE study were not representative of every patient with schizophrenia. In particular, they were all willing to enter a medication trial and were therefore all seeking treatment. In addition, first-episode and treatment-refractory patients were excluded, while they may be at higher risk for violent behavior^13^. Nevertheless, only a small minority of screened patients were excluded for these reasons (7%) and the study included a very large number of sites to ensure representativeness of the US schizophrenia population.

In conclusion, this innovative study used data from the CATIE trials to create cross-lag models and to investigate the longitudinal relationship between persistent cannabis use and violence within a very large schizophrenia cohort. In doing so, a unidirectional longitudinal relationship between cumulative cannabis use and subsequent violent behaviors was found, thereby confirming our initial hypothesis. These findings are consistent with what was previously shown in populations with various severe mental illnesses. As cannabis is an important risk factor for violence in the schizophrenia population, its consumption should be considered separately from that of other drugs when assessing and managing risks in clinical and in legal settings. As an increasingly significant body of literature suggests that a link exists between cannabis use and violence in people with severe mental illnesses, further studies should investigate the underlying mechanisms of this association.

## Methods

### Study design and sample characteristics

**Fig. 1:**
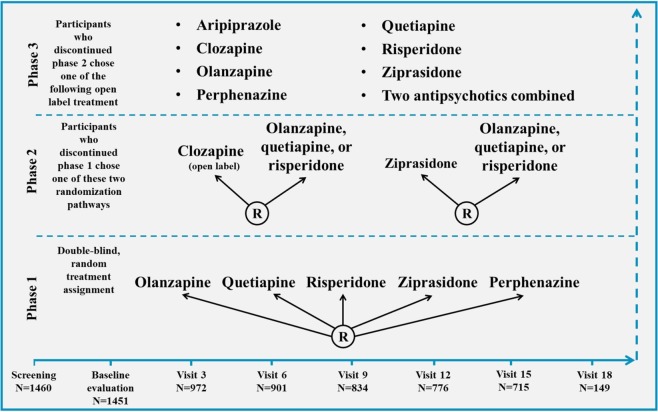
CATIE schizophrenia trial design. Responders stayed on assigned medication for up to 18 months. Follow-up visits occurred every 3 months, whereas phases had no predetermined duration. Phase 1 also included two subphases, named 1A and 1B. Phase 1A: participants with tardive dyskinesia were not randomized to perphenazine. Phase 1B: participants who failed to respond to perphenazine were randomized to olanzapine, quetiapine, or risperidone before becoming eligible for phase 2. R, randomization. This figure was adapted from Stroup et al.^46^.

Participants included in this study were all part of the CATIE project, a large clinical trial conducted by the National Institute of Mental Health between December 2000 and December 2004. This study comprised 1460 patients who currently meet or have met in the past DSM-IV diagnostic criteria for schizophrenia, based upon the Structured Clinical Interview for DSM-IV^45^. It was approved by the institutional review board at each site and the patients or their legal guardians provided their written informed consent. The detailed study description and design can be found elsewhere^46^. In addition, the study design is described in Fig. 1 and the study characteristics, including inclusion and exclusion criteria, are detailed in Supplementary Table 1.

The present study reports findings over 18 months, divided into three 6-month periods encompassing the baseline visit and four follow-up visits (at 3, 6, 9, and 12 months post baseline). All participants with a valid follow-up visit 6 and/or 12 were included (*N* = 965).

### Assessments

**Fig. 2:**
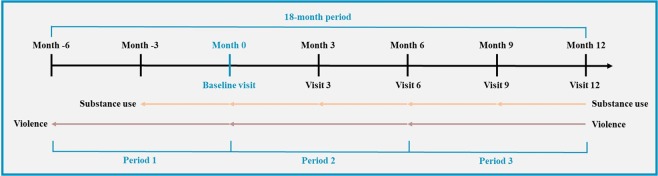
Timeline of the retrospective measurements of violence and substance use. Substance use was assessed every three months, whereas violence was assessed every 6 months. This study covered an 18-month period, from 6 months before the baseline to 12 months after the baseline. Visits 3 to 12: follow-up visits, taking place every 3 months. The three 6-month periods have been used for our analyses.

Both the dependent variable (violence) and the independent variable (cannabis use) were measured at baseline, and again at specific follow-ups. Substance use was assessed at 3-month intervals: participants were questioned on their substance use within the preceding 3 months. Conversely, violence was measured every 6 months. Violent behaviors within the 6 months prior to their visit were assessed. To simplify our analyses, the timeline was divided into three 6-month periods. The first period evaluated violence occurring 6 months before the baseline and substance use 3 months before the baseline. The second period included substance use and violence occurring within the first 6 months of the study and the third period included substance use and violence within the following 6 months. This timeline is schematized in Fig. 2.

**Fig. 3:**
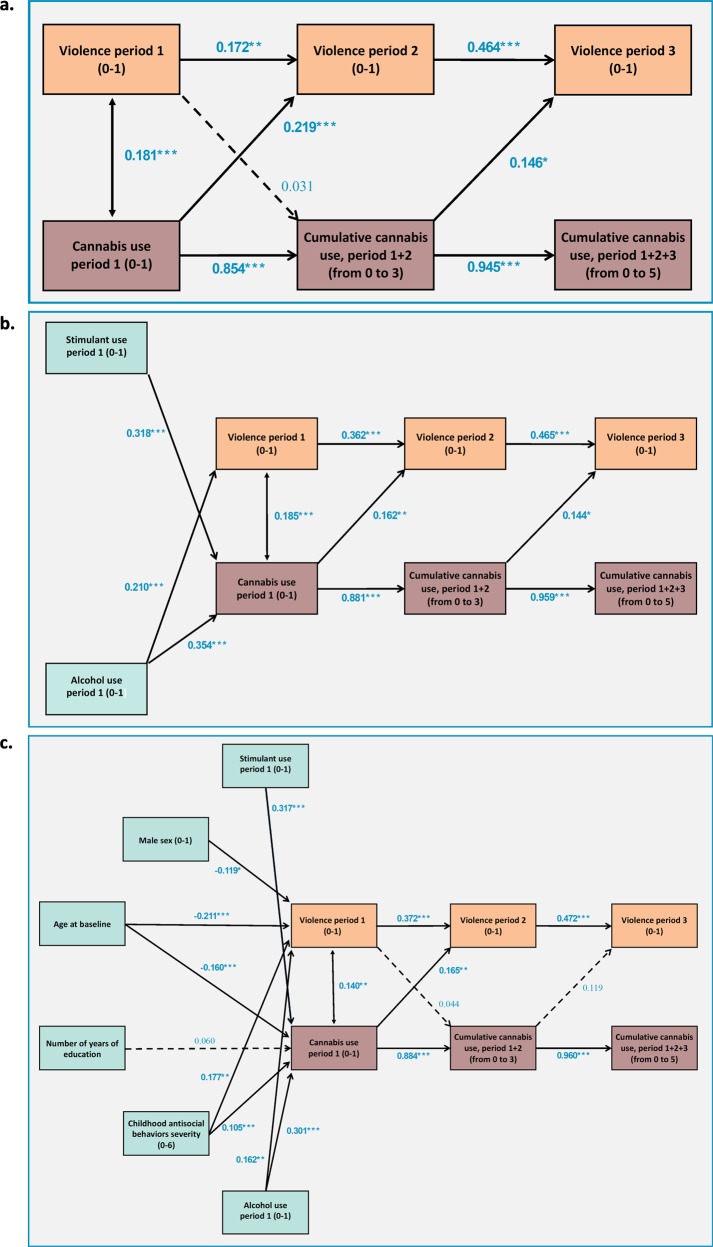
Standardized coefficients representing the association between persistent cannabis use and violence across time. **a** Cross-lag model 1, without covariables. **b** Cross-lag model 2, controlled for stimulant and alcohol use during the period 1. **c** Cross-lag model 3, adjusted for sex, age, educational level, childhood antisocial behaviors, stimulant use, and alcohol use, assessed during the baseline interview. Full lines: statistically significant associations (*p* < 0.05). Dotted lines: not statistically significant associations. Only associations with a *p*-value under 0.10 were presented. *p < 0.05, **p < 0.01, ***p < 0.001. *N* = 965.

Violent behaviors were assessed every 6 months (20 weeks) and were self-reported using a shortened version of the MacArthur Abbreviated Community Violence Instrument, initially developed for the MacArthur Violence Risk Assessment Study^47^. This questionnaire assessed aggressive behaviors within the 6 months preceding its completion. Such behaviors include violence towards others (e.g., hitting someone, trying to force someone to have sex against his/her will, using a lethal weapon against someone) and threats of serious violence (e.g., threatening someone with a lethal weapon). Nine items assessed violent behaviors and nine others assessed the subject’s victimization. This information was validated with a Family/Caregiver interview throughout which the following question was asked: “During the past 6 months, was (client’s name) involved in any physical fights?*”* However, information provided from a collateral relating to violence was only available for 45.3% of the sample for period 1, 34.1% for period 2, and 28.8% for period 3.

In this study, participants were classified into two categories for each 6-month period: nonviolent (those who did not commit any acts of violence) and violent (those who committed at least one violent act). If a participant did not report any violence, but his/her collateral claimed otherwise, the participant was classified into the violent group. This adjustment occurred in 2.9% of participants for the period 1, 1.2% for the period 2, and 0.9% for the period 3.

As for substance use, the consumption of cannabis, alcohol, cocaine, opiates, PCP, amphetamines, and tobacco was self-reported every 3 months (10 weeks) in the General Clinical Status interview with the question, “In the past three months, have you used [substance]?” The participant was attributed a score of 1 if s/he had consumed cannabis during the period assessed and was attributed a score of 0 if s/he had not. Three variables (one for each period) were then created to represent the cumulative cannabis use:

- The first variable represents cannabis use during period 1, within the 3 months preceding the baseline interview (0–1);
- The second variable represents the cumulative use of cannabis during periods 1 and 2, which corresponds to the number of visits during which the participant reported cannabis consumption within the previous 3 months (range between 0 and 3);
- The third variable represents the cumulative use of cannabis during periods 1, 2, and 3 (range between 0 and 5).

To account for some of the participants’ missing time points (see Supplementary Table 2), the mean of the other time points for those participants was used to estimate the total amount of time points. Cannabis, alcohol, cocaine, opiates, PCP, and amphetamine use were only considered for the 3-month period preceding the baseline, when the prevalence was the highest (0–1). A “stimulant” variable, combining cocaine and amphetamine use, was created to yield a larger number of participants for this group and a higher statistical power.

Possibly confounding variables were selected based on theoretical assumptions of violence predictors in the existent literature. Such predictors include psychiatric diagnoses, sociodemographic variables and treatment history, childhood problem behaviors, symptom severity, quality of life, insight and treatment attitude, antipsychotic treatment, and medication adherence. These measures are described in Supplementary Table 3.

### Statistical analyses

To begin, descriptive analyses were conducted using SPSS Statistics 25^48^ to select pertinent covariables to include in the models. Potential violence predictors were selected based on the existent literature^13^. The association between every pertinent baseline measurement and violence at 6 months was calculated using the most appropriate descriptive test (Mann–Whitney tests for continuous variables, and *χ*^2^ (more than five participants expected in cell) or Fisher tests (when at least one cell had an expected count of less than 5) for dichotomic variables).

In addition, cross-lag models were conducted using MPLUS version 8^49^, to evaluate the association between cannabis use and violence across time. Three models were estimated:

1. Association between persistent cannabis use and violence across time, without covariables;
2. Association between persistent cannabis use and violence across time, with alcohol and stimulant use at baseline as covariables;
3. Association between persistent cannabis use and violence across time, with all baseline measurements significantly associated to violence at 6 and/or 12 months as covariables.

Standardized model estimates and their reporting two-tailed *p*-values were outlined schematically. The threshold for a statistically significant *p*-value was set at 0.05.

## Supplementary information

Supplementary material

Reporting Sum

## Data Availability

The Clinical Antipsychotic Trials of Intervention Effectiveness Schizophrenia Trial is a limited access dataset available on request, under certain conditions, from National Institutes of Mental Health clinical trials.
