# Supplementary material for: Persistent cannabis use as an independent risk factor for violent behaviors in patients with schizophrenia
Source: NPJ Schizophr. 2020 May 11;6:14. doi: 10.1038/s41537-020-0104-x (PMC7214412; doi:10.1038/s41537-020-0104-x)
Supplement: Supplementary file 1 — Supplementary material [file 41537_2020_104_MOESM1_ESM.pdf]

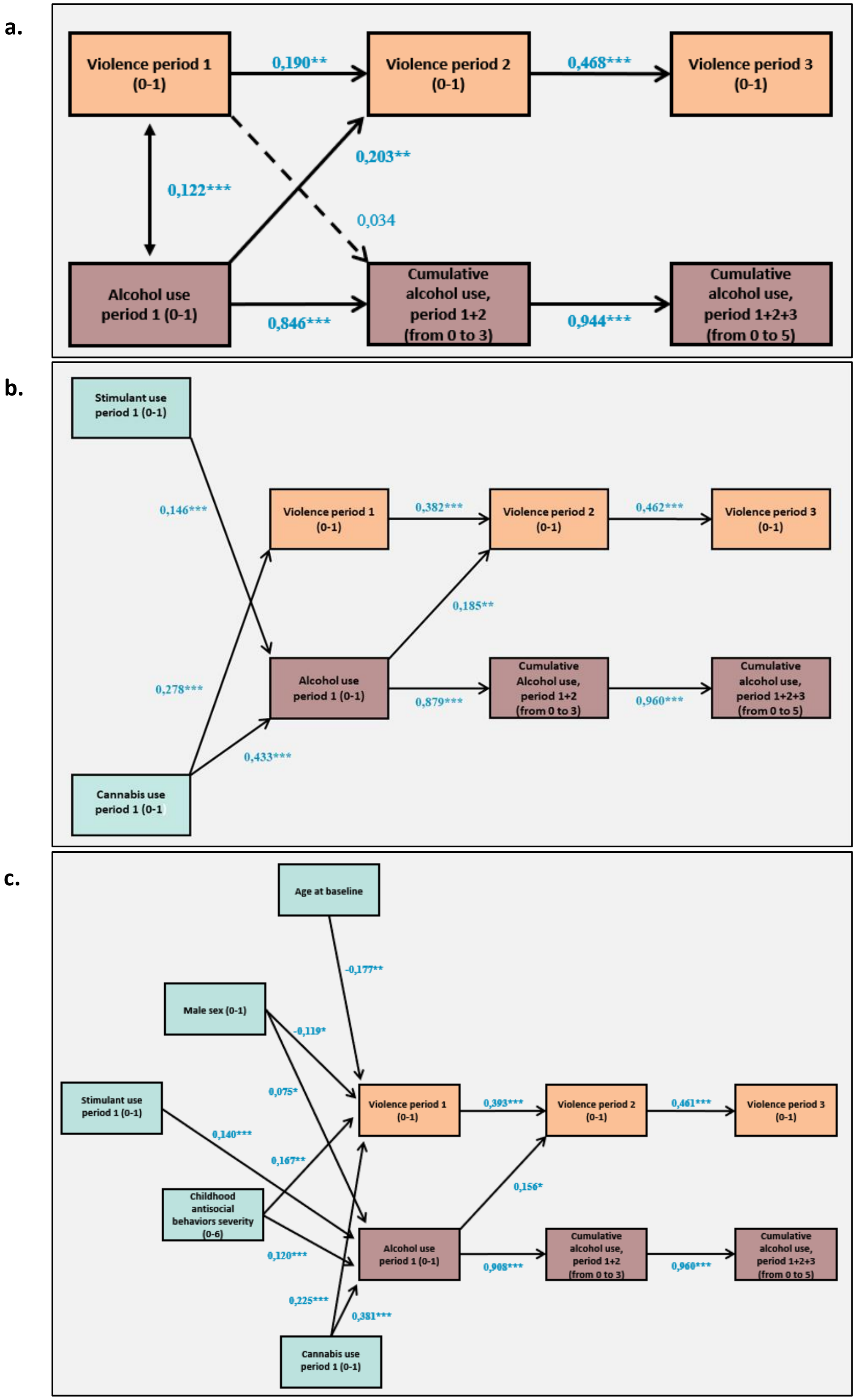


**Supplementary Figure 1. Standardized coefficients representing the association between persistent alcohol use and violence across time. a.** Cross-lag model 4, without covariables. **b.** Cross-lag model 5, controlled for stimulant and cannabis use during the period 1. **c.** Cross-lag model 6, adjusted for sex, age, educational level, childhood antisocial behaviors, stimulant use and cannabis use, assessed during the baseline interview. Full lines: statistically significant associations (p <0.05). Dotted lines: not statistically significant associations. Only associations with a p-value under 0.10 were presented. *p< 0.05, **p< 0.01, ***p< 0.001. N=965

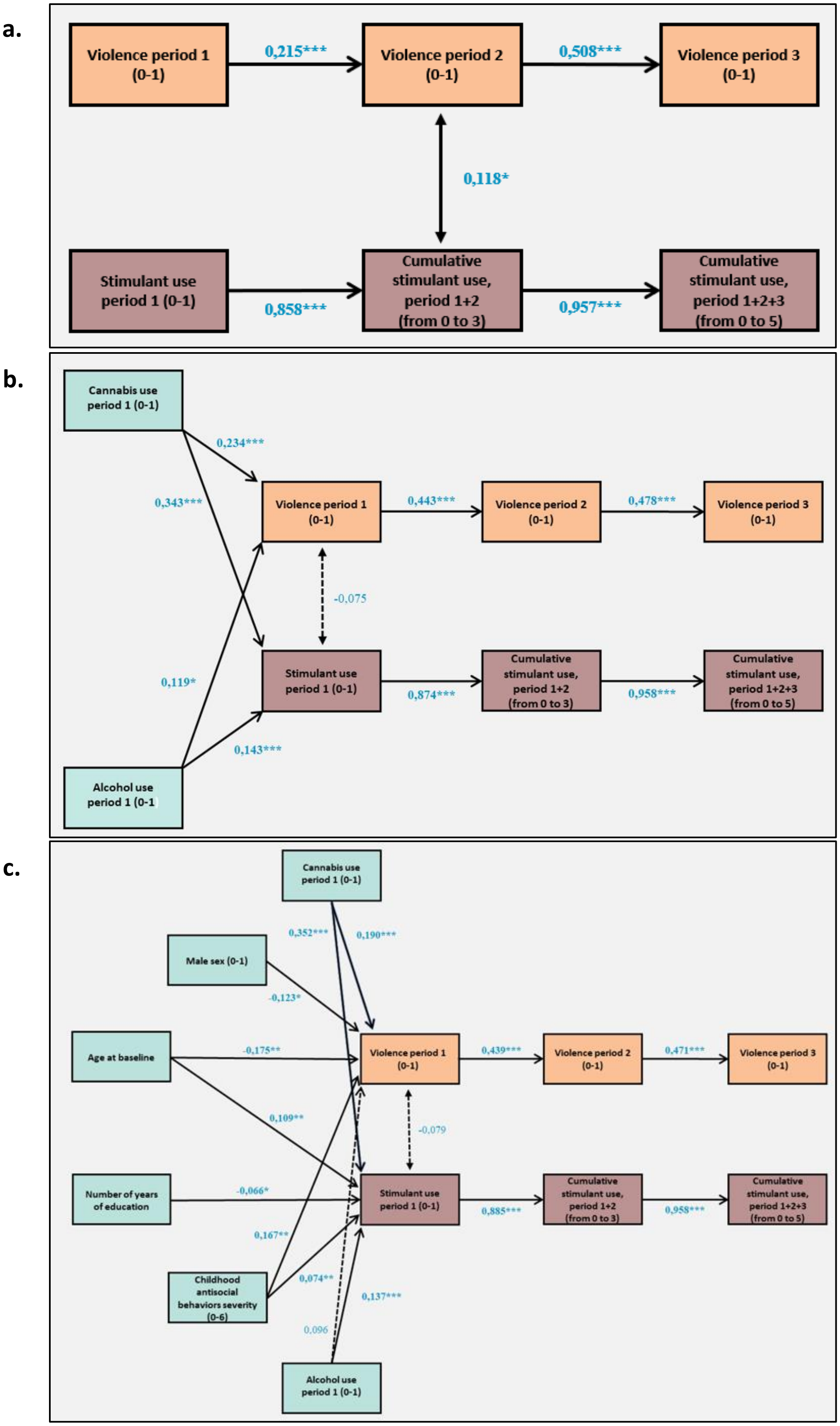


**Supplementary Figure 2. Standardized coefficients representing the association between persistent stimulant use and violence across time. a.** Cross-lag model 7, without covariables. **b.** Cross-lag model 8, controlled for alcohol and cannabis use during the period 1. **c.** Cross-lag model 9, adjusted for sex, age, educational level, childhood antisocial behaviors, alcohol use and cannabis use, assessed during the baseline interview. Full lines: statistically significant associations ($p < 0.05$). Dotted lines: not statistically significant associations. Only associations with a p-value under 0.10 were presented. *$p < 0.05$, **$p < 0.01$, ***$p < 0.001$. N=965

**Supplementary Table 1. Study Characteristics of the Clinical Antipsychotic Trials of Intervention Effectiveness.**

| Study Characteristics | Description |
|---|---|
| **Study period** | Between December 2000 and December 2004. |
| **Study sites** | 54 clinical sites, representing:<br>• 24 states of the United-States;<br>• 48 cities and towns.<br><br>Including:<br>• 16 university clinics;<br>• 14 mixed-system sites;<br>• 10 state mental health agencies;<br>• 7 Veterans' Affairs Medical Centres;<br>• 6 private non-profit centers;<br>• 4 private practice sites. |
| **Sample size** | From 1493 recruited participants:<br>• 1460 completed the baseline evaluation;<br>• 1432 received at least one dose of antipsychotic medication;<br>• 1061 discontinued the study medication before 18 months;<br>• 965 had a valid follow-up visit 6 and/or 12. |
| **Primary aim** | To compare the effectiveness of some antipsychotic medications used to treat schizophrenia (conventional vs. atypical) in a representative sample of patients seeking treatment for chronic schizophrenia. |
| **Intervention** | Participants were first randomized to one medication (olanzapine, quetiapine, risperidone, ziprasidone, or perphenazine). In the case of treatment failure, they were re-randomized to a new treatment (one of the above, clozapine, aripiprazole, fluphenazine decanoate, or two medications combined). |
| **Follow-ups** | Follow-up interviews occurred:<br>• Every 10 weeks, from baseline to 18-months post-baseline;<br>• At the end of each phase (1a, 1b, 2, 3). |
| **Inclusion and exclusion criteria** | Inclusion criteria:<br>• 18-65 years of age;<br>• Meet or have met the DSM-IV criteria for schizophrenia;<br>• Have a condition appropriate for treatment with an oral medication;<br>• Demonstrate adequate decisional capacity to provide informed consent.<br><br>Exclusion criteria:<br>• Have a DSM-IV diagnosis of:<br>  o Schizoaffective disorder;<br>  o Mental retardation;<br>  o Pervasive developmental disorder;<br>  o Delirium;<br>  o Dementia;<br>  o Amnesia;<br>  o Other cognitive disorders;<br>• Have a well documented, drug related, serious adverse reaction or having a history of failure to respond to one of the proposed treatment arms;<br>• Have a first episode of schizophrenia;<br>• Have been treated with clozapine for treatment resistance;<br>• Have a medical or pharmacological contraindication to any of the drugs to which they might be assigned. |

**Supplementary Table 2. Sample Characteristics.**

| Baseline Characteristics | Mean | Standard Deviation | Range |
|---|---|---|---|
| Age, N=964 | 41.1 | 11.0 | 18 - 67 |
| Education years, N=960 | 11.6 | 3.5 | 1 - 21 |
| Years since first treatment, N=937 | 16.5 | 11.3 | 0 - 56 |
| Years since first prescribed antipsychotic medication, N=942 | 14.2 | 11.0 | 0 - 56 |
| Number of previous hospitalizations, lifetime, N=964 | 2.7 | 1.5 | 0 - 4 |
| Number of previous hospitalizations, past year, N=965 | 0.6 | 0.9 | 0 - 4 |
| | **N** | **%** | |
| Male gender, N=965 | 698 | 72.3 | |
| Did not complete high school, N=965 | 241 | 25.0 | |
| Married, N=96**4** | 107 | 11.1 | |
| Veterans, N=964 | 204 | 21.1 | |
| Employed full-time, N=956 | 60 | 6.3 | |
| **Ethnicity, N=965** | | | |
| White | 602 | 62.4 | |
| Black | 326 | 33.8 | |
| American Indian or Alaska Native | 15 | 1.6 | |
| Asian | 27 | 2.8 | |
| Hispanic Latino or Spanish Origin | 111 | 11.5 | |
| Hawaiian or Pacific Islander | 7 | 0.7 | |
| **Comorbid psychiatric diagnoses, N=965** | | | |
| Obsessive-compulsive disorder | 43 | 4.5 | |
| Other anxiety disorder | 86 | 8.9 | |
| Major depression | 135 | 14.0 | |
| Alcohol dependence | 84 | 8.7 | |
| Alcohol abuse | 85 | 8.8 | |
| Drug dependence | 73 | 7.6 | |
| Drug abuse | 112 | 11.6 | |
| Antisocial personality disorder | 7 | 0.7 | |
| Other personality disorder | 10 | 1.0 | |
| Other comorbid diagnosis | 38 | 3.9 | |
| No comorbid condition | 571 | 59.2 | |

**Supplementary Table 2. Sample Characteristics.** (continued)

| Dynamic Characteristics | Period 1, N=906 | | Period 2, N=861 | | | | Period 3, N=751 | | | |
|---|---|---|---|---|---|---|---|---|---|---|
| | N | % | N | | % | | N | | % | |
| At least 1 act of violence | 105 | 11.5 | 52 | | 6.0 | | 40 | | 5.3 | |
| At least 1 self-reported act of severe violence[a] | 40 | 4.4 | 21 | | 2.4 | | 10 | | 1.3 | |
| At least 1 self-reported other aggressive act[b] | 50 | 5.5 | 23 | | 2.7 | | 21 | | 2.8 | |
| | Period 1, N=962 | | Periods 1 and 2, N=965 | | | | Periods 1, 2 and 3, N=965 | | | |
| | Mean | SD | Mean | | SD | | Mean | | SD | |
| Cumulative cannabis consumption | 0.2 | 0.4 | 0.4 | | 0.9 | | 0.6 | | 1.4 | |
| | Baseline N=962 | | Visit 3 N=840 | | Visit 6 N=892 | | Visit 9 N=817 | | Visit 12 N=768 | |
| | N | % | N | % | N | % | N | % | N | % |
| Cannabis consumption | 154 | 16.0 | 98 | 11.7 | 110 | 12.3 | 94 | 11.5 | 81 | 10.5 |
| Alcohol consumption | 334 | 34.7 | 282 | 28.8 | 248 | 27.8 | 236 | 28.9 | 210 | 27.3 |
| Cocaine consumption | 64 | 6.7 | 46 | 5.5 | 36 | 4.0 | 42 | 5.1 | 36 | 4.7 |
| Amphetamine consumption | 15 | 1.6 | 8 | 1.0 | 12 | 1.3 | 9 | 1.1 | 6 | 0.8 |
| Opiate consumption | 9 | 0.9 | 3 | 0.4 | 13 | 1.5 | 5 | 0.6 | 6 | 0.8 |
| PCP consumption | 1 | 0.1 | 0 | 0.0 | 1 | 0.1 | 1 | 0.1 | 0 | 0.0 |
| Other drug consumption | 9 | 0.9 | 5 | 0.6 | 3 | 0.3 | 5 | 0.6 | 4 | 0.5 |
| Medication characteristics | | | Proportion of the sample, N=965 | | | | | | | |
| | | | N | | | | % | | | |
| Quetiapine exposure, from baseline to visit 12 | | | 369 | | | | 38.2 | | | |
| Clozapine exposure, from baseline to visit 12 | | | 81 | | | | 8.4 | | | |
| Non-adherence to medication | | | 170 | | | | 17.6 | | | |

a. Severe violence: a battery that resulted in physical injury, sexual assaults, assaultive acts that involved the use of a weapon, or threats made with a weapon in hand.
b. Other aggressive acts: a battery that did not result in physical injury.

**Supplementary Table 3. Baseline Covariable Measurements.**

| Covariables | Measurement instrument | Characteristics | References |
|---|---|---|---|
| Psychiatric diagnoses | *Structured Clinical Interview for DSM-IV, Axis I disorders* (SCID-I) | This semi-structured interview is a widely used tool for assessing psychiatric diagnoses. It was used during the screening interview to confirm the schizophrenia diagnosis, but also to assess other comorbid diagnoses: obsessive-compulsive disorder, other anxiety disorders (post-traumatic stress disorder, panic disorder, agoraphobic without a history of panic disorder, social phobia, and specific phobia), major depression, alcohol dependence and drug dependence. | 1 |
| Sociodemographic variables and treatment history | The interviewer questioned the participant to obtain sociodemographic information (such as sex, age, educational level, ethnicity, marital status, residential status), treatment history and course of illness (including information on previous hospitalizations, treatments, and antipsychotic medications). | | N/A |
| Childhood problem behaviors | Childhood antisocial behaviors were assessed using a set of six questions of the SCID-IV, which evaluates problematic behaviors before the age of 15, such as “Did you run away from home overnight more than once before age 15?” Severity, ranging from 0 to 6, was determined based on the number of “yes” answers. | | N/A |
| Psychotic symptomatology | *Positive and Negative Syndromes Scale* (PANSS) | This 30-item semi-structured interview assesses positive, negative and general symptoms of psychopathology. Each item is rated from 1 (absent) to 7 (extreme). The PANSS has a widespread use in clinical psychosis studies, and its reliability in assessing psychopathology across diverse patient populations is well demonstrated. | 2, 3 |
| Depressive symptoms | *Calgary Depression Rating scale* (CDRS) | The CDRS is a 9-item scale designed for use with schizophrenia patients. It was shown to be reliable within this population. | 4, 5 |
| Quality of life | *Heinrichs-Carpenter Quality of Life Scale* (QLS) | Social functioning, interpersonal relationships and intrapsychic well-being were assessed using the QLS, a well-validated clinician-rated scale which is widely used in clinical trials with schizophrenia patients. | 6 |
| Insight and treatment attitude | *Insight into Treatment Attitude Questionnaire* (ITAQ) | This 11-item scale was designed to measure awareness of illness and treatment-need insight in schizophrenia patients. Each item consists of a question and is scored 0–1. | 7 |
| Antipsychotic treatment | Quetiapine and clozapine exposure were considered in our study.<br>Indeed, a study using baseline data from the same database found quetiapine to be less efficient for violence reduction, while perphenazine, risperidone, olanzapine and ziprasidone were found to be equivalently efficient. Additionally, clozapine, another antipsychotic included later in the CATIE study, could be more useful than other antipsychotics to prevent violence. | | 8, 9 |
| Medication adherence | The non-adherence to treatment between the baseline interview and visit 12 was assessed by the question, “Since the last study visit, has the patient been taking the medication using the dosing frequency assigned by QTONE?” (QTONE being the patient randomization system). This question was answered by the interviewer, using his/her clinical judgment, based on collected information (proportion of capsules taken and information obtained from patients using the *Medication Adherence Form*). | | N/A |

**References**


1. First, M.B., Spitzer, R.L., Gibbon, M., Williams, J.B., 1997. User's guide for the Structured clinical interview for DSM-IV axis I disorders SCID-I: clinician version. American Psychiatric Pub.
2. Kay, S.R., Fiszbein, A., Opler, L.A., 1987. The positive and negative syndrome scale (PANSS) for schizophrenia. Schizophrenia bulletin 13(2), 261-276.
3. Opler, M.G.A., Yavorsky, C., Daniel, D.G., 2017. Positive and Negative Syndrome Scale (PANSS) Training: Challenges, Solutions, and Future Directions. Innovations in clinical neuroscience 14(11-12), 77-81.
4. Addington, D., Addington, J., Schissel, B., 1990. A depression rating scale for schizophrenics. Schizophrenia research 3(4), 247-251.
5. Addington, D., Addington, J., Maticka-Tyndale, E., Joyce, J., 1992. Reliability and validity of a depression rating scale for schizophrenics. Schizophrenia research 6(3), 201-208.
6. Heinrichs, D.W., Hanlon, T.E., Carpenter, W.T., Jr., 1984. The Quality of Life Scale: an instrument for rating the schizophrenic deficit syndrome. Schizophrenia bulletin 10(3), 388-398.
7. McEvoy, J.P., Freter, S., Everett, G., Geller, J.L., Appelbaum, P., Apperson, L.J., Roth, L., 1989. Insight and the clinical outcome of schizophrenic patients. The Journal of nervous and mental disease 177(1), 48-51.
8. Swanson, J.W., Swartz, M.S., Van Dorn, R.A., Volavka, J., Monahan, J., Stroup, T.S., McEvoy, J.P., Wagner, H.R., Elbogen, E.B., Lieberman, J.A., 2008. Comparison of antipsychotic medication effects on reducing violence in people with schizophrenia. The British journal of psychiatry : the journal of mental science 193(1), 37-43.
9. Patchan, K., Vyas, G., Hackman, A.L., Mackowick, M., Richardson, C.M., Love, R.C., Wonodi, I., Sayer, M.A., Glassman, M., Feldman, S., Kelly, D.L., 2018. Clozapine in Reducing Aggression and Violence in Forensic Populations. The Psychiatric quarterly 89(1), 157-168.

**Supplementary Table 4. Cross-lag Models 1, 2 and 3 – Standardized Coefficients, Standard Errors and their Reporting p-Values.**

| Variable 1 | Test | Variable 2 | Standardized coefficients | Standard errors | Two-tailed p-value |
|---|---|---|---|---|---|
| **Cross-lag model 1** | | | | | |
| Violence period 3 | ON | Violence period 2 | 0.464 | 0.117 | < 0.001*** |
| | | Cannabis periods 1+2 | 0.146 | 0.070 | 0.038* |
| Violence period 2 | ON | Violence period 1 | 0.172 | 0.055 | 0.002** |
| | | Cannabis periods 1 | 0.219 | 0.053 | < 0.001*** |
| Cannabis periods 1+2+3 | ON | Cannabis periods 1+2 | 0.945 | 0.006 | < 0.001*** |
| | | Violence period 2 | -0.020 | 0..018 | 0.27 |
| Cannabis periods 1+2 | ON | Cannabis period 1 | 0.854 | 0.010 | < 0.001*** |
| | | Violence period 1 | 0.031 | 0.017 | 0.074 |
| Violence period 1 | WITH | Cannabis period 1 | 0.181 | 0.028 | < 0.001*** |
| Violence period 2 | WITH | Cannabis periods 1+2 | -0.015 | 0.059 | 0.80 |
| Violence period 3 | WITH | Cannabis periods 1+2+3 | 0.008 | 0.073 | 0.91 |
| **Cross-lag model 2** | | | | | |
| Violence period 3 | ON | Violence period 2 | 0.465 | 0.116 | < 0.001*** |
| | | Cannabis periods 1+2 | 0.144 | 0.072 | 0.046* |
| Violence period 2 | ON | Violence period 1 | 0.362 | 0.092 | < 0.001*** |
| | | Cannabis periods 1 | 0.162 | 0.060 | 0.007** |
| Cannabis periods 1+2+3 | ON | Cannabis periods 1+2 | 0.959 | 0.006 | < 0.001*** |
| | | Violence period 2 | -0.026 | 0.016 | 0.12 |
| Cannabis periods 1+2 | ON | Cannabis period 1 | 0.881 | 0.013 | < 0.001*** |
| | | Violence period 1 | 0.034 | 0.025 | 0.18 |
| Violence period 1 | WITH | Cannabis period 1 | 0.185 | 0.047 | < 0.001*** |
| Violence period 2 | WITH | Cannabis periods 1+2 | -0.095 | 0.079 | 0.23 |
| Violence period 3 | WITH | Cannabis periods 1+2+3 | 0.027 | 0.083 | 0.75 |
| Violence period 1 | ON | Alcohol period 1 | 0.210 | 0.049 | < 0.001*** |
| | | Stimulants period 1 | 0.014 | 0.047 | 0.76 |
| Cannabis period 1 | ON | Alcohol period 1 | 0.354 | 0.027 | < 0.001*** |
| | | Stimulants period 1 | 0.318 | 0.020 | < 0.001*** |

**Supplementary Table 4. Cross-lag Models 1, 2 and 3 – Standardized Coefficients, Standard Errors and their Reporting p-Values.** (continued)

| Variable 1 | Test | Variable 2 | Standardized coefficients | Standard errors | Two-tailed p-value |
|---|---|---|---|---|---|
| **Cross-lag model 3** | | | | | |
| Violence period 3 | ON | Violence period 2 | 0.472 | 0.104 | < 0.001*** |
| | | Cannabis periods 1+2 | 0.119 | 0.069 | 0.08 |
| Violence period 2 | ON | Violence period 1 | 0.372 | 0.088 | < 0.001*** |
| | | Cannabis periods 1 | 0.165 | 0.060 | 0.006** |
| Cannabis periods 1+2+3 | ON | Cannabis periods 1+2 | 0.960 | 0.006 | < 0.001*** |
| | | Violence period 2 | -0.024 | 0.017 | 0.15 |
| Cannabis periods 1+2 | ON | Cannabis period 1 | 0.884 | 0.012 | < 0.001*** |
| | | Violence period 1 | 0.044 | 0.024 | 0.07 |
| Violence period 1 | WITH | Cannabis period 1 | 0.140 | 0.047 | 0.003** |
| Violence period 2 | WITH | Cannabis periods 1+2 | -0.116 | 0.081 | 0.15 |
| Violence period 3 | WITH | Cannabis periods 1+2+3 | -0.018 | 0.093 | 0.85 |
| Violence period 1 | ON | Alcohol period 1 | 0.162 | 0.051 | 0.002** |
| | | Stimulants period 1 | 0.015 | 0.045 | 0.75 |
| | | Male sex | -0.119 | 0.053 | 0.026* |
| | | Age | -0.211 | 0.053 | < 0.001*** |
| | | Education level | -0.043 | 0.051 | 0.41 |
| | | Childhood antisocial behaviors | 0.177 | 0.053 | 0.001** |
| Cannabis period 1 | ON | Alcohol period 1 | 0.301 | 0.027 | < 0.001*** |
| | | Stimulants period 1 | 0.317 | 0.020 | < 0.001*** |
| | | Male sex | < 0.001 | 0.034 | 0.11 |
| | | Age | -0.160 | 0.029 | < 0.001*** |
| | | Education level | 0.060 | 0.031 | 0.05 |
| | | Childhood antisocial behaviors | 0.105 | 0.026 | < 0.001*** |

* p< 0.05; ** p< 0.01; *** p< 0.001.
